# The publication quality of laboratory values in clinical studies in neonates

**DOI:** 10.1038/s41390-022-02385-1

**Published:** 2022-12-22

**Authors:** Karel Allegaert, Heidrun Hildebrand, Kanwaljit Singh, Mark A. Turner

**Affiliations:** 1 Department of Pharmaceutical and Pharmacological Sciences, KU Leuven, Leuven, Belgium; 2 Department of Development and Regeneration, KU Leuven, Leuven, Belgium; 3 Leuven Child and Youth Institute, KU Leuven, Leuven, Belgium; 4 Department of Hospital Pharmacy, Erasmus MC, Rotterdam, the Netherlands; 5 Pediatric Development, Research & Development, Pharmaceuticals, Bayer AG, Berlin, Germany; 6 International Neonatal Consortium, Critical Path Institute, Tucson, AZ USA; 7 Institute of Lifecourse and Medical Sciences, University of Liverpool, Liverpool Health Partners, Liverpool, UK; 8 Centre for Women’s Health Research, Liverpool Women’s Hospital, Crown Street, Liverpool, L8 7SS UK

## Abstract

**Background:**

There are no generally accepted age-appropriate reference ranges for laboratory values in neonates. This also matters for drug development. The International Neonatal Consortium (INC) is engaged to define actionable reference ranges of commonly used laboratory values in neonates.

**Methods:**

A structured literature search was performed to identify standards or recommendations for publications that present neonatal laboratory data to assess the publication quality of laboratory values in neonates. Using a modified Delphi approach, an assessment and data extraction instrument to screen on completeness of information was developed.

**Results:**

On 2908 hits, 281 papers were retained for full reading and 257 for data extraction. None of the papers reported a publication standard. Using the extraction instrument, most papers presented single country or unit findings. The median number of neonates was 120, with uncertainty on single or repeated measurements. Clinically meaningful information on age, sex, and medical conditions was commonly provided. Information on pharmacotherapy, equipment, analytical method, or laboratory location was rarely mentioned.

**Conclusions:**

Published information on laboratory values for neonates is sparse, not systematic, and incomplete. This undermines efforts to compare treatments, safety monitoring, or clinical management. Furthermore, there appears to be no standard yet to report laboratory values in neonates.

**Impact:**

- There are no generally accepted age-appropriate reference ranges for laboratory values in neonates, leading to a significant knowledge gap, also for safety reporting and drug development in neonates.
- We performed a literature search to identify standards or recommendations for publications on neonatal laboratory data and to assess the publication quality of laboratory values in clinical studies involving neonates.
- Standards or recommendations for publications that present neonatal laboratory data were not identified, while published information on laboratory values for neonates is sparse, not systematic, and incomplete.

## Introduction

A laboratory result report can be defined as “*a combination of specimen information and results … and contains other information pertinent to correct interpretation*”.^1^ In clinical studies, laboratory values are used to detect and quantify (side) effects or as exclusion/inclusion criteria. However, this relies heavily on availability of age-appropriate reference ranges and normative values.^1,2^ During neonatal research, disentangling “true” effects from confounders (e.g., maturational changes in laboratory values, organ dysfunction, co-morbidities) remains challenging. Therefore, neonatal laboratory reference ranges (*information pertinent to correct interpretation*) are particularly important.^1,2^ Unfortunately, there are no generally accepted age-appropriate reference ranges or actionable thresholds in neonates, especially for preterm infants. This has been identified as a critical knowledge gap, since laboratory values are commonly based on ad hoc local data or even adult reference values.^2^

Although multiple factors contribute to difficulties with safety reporting and neonatal drug development, this paradigm is changing.^3^ For example, the Critical Path Institute’s International Neonatal Consortium (INC) developed the Neonatal Adverse Event Severity Scale (NAESS).^4^ However, when the NAESS was developed, laboratory-based adverse events were not included because of absence of age-appropriate reference values.

INC is currently working toward a data aggregation and integration effort to define actionable reference ranges of commonly used laboratory values in neonates.^5^ These reference values will be derived from real world data (RWD) and presented transparently. INC further aims to present the data quality and analysis. To inform how the quality of reference values is presented, a structured literature search was performed in order to:

1. Identify standards for publications that present neonatal laboratory data.
2. Assess the publication quality of laboratory values presented in clinical studies in neonates.

It is hereby important to note that standards or recommendations for publications that present laboratory data are different from standards that inform how laboratories report to clinicians. Furthermore, we are fully aware that laboratory values are also relevant in disease diagnosis and prognosis.

## Methods

To create an overview on the quality of presenting laboratory values in scientific publications, a structured search and questionnaire to screen on information completeness were developed.

### Search strategy

A search strategy was created and subsequently performed on May, 20 2021 by Simone Grum (librarian, simone.grum@bayer.com) (Embase and Medline, search strings provided in Supplement 1).

### Development of a screening questionnaire and a data extraction document

The questionnaire was developed through an adapted Delphi. In a first step, 4 core members (K.A., H.H., K.S., M.A.T.) of the INC-RWD group discussed common analytes to consider, population to target, and data to be extracted in consecutive online meetings (June 2021). This proposal was subsequently discussed in the broader INC RWD group (online) on June 23, 2021 for confirmation and adaptations.

### Data extraction

The final questionnaire was first pilot tested in the first 20 papers to examine feasibility of data extraction by the same core members. Abstracts were assessed on the presence of laboratory values datasets or any reference range (or control values) in the relevant age category, with focus on the analytes of interest by one of the 4 core members. Following this pilot, the structured data extraction was subsequently performed by two researchers (K.A., and Mado Bangia, student-employee to KU Leuven for this project).

## Results

### Search and screening tool

Based on this search strategy, 2908 hits were retained for screening after duplicate removal. The final screening tool focused on *commonly used laboratory values* [(Hemoglobin; Red Blood Cells—Erythrocytes; Leukocytes; Platelets; Sodium; Potassium; Calcium; Phosphorus; Glucose; Creatinine; Blood urea nitrogen (BUN); Bilirubin; Aspartate aminotransferase (AST); Alanine aminotransferase (ALT); Alkaline phosphatase (ALP); C-reactive protein (CRP); Procalcitonin (PCT), and *populations* (neonates, day 0–28 days, infants, >28 days to 1 year)]. Using these parameters, 281 papers were retained for full reading.

### Data extraction

The final data extraction tool agreed within the INC-RWD group included the origin of data (countries), number of sites involved, number of neonates with at least one measurement, number of neonates with repeated measurements, demographics (gestational age range and subgroups included, sex, age at sampling, medical conditions, subgroups, and drugs administered), laboratory values (analytes: numbers, range, and units for each analyte), information on equipment and methodology as provided (including in-hospital, external laboratory, or point-of-care testing), out/inpatients, analysis method, statistical method (range, centiles or standard deviation scores, (non)-parametric), the presence of a validation set or effort, the availability of access to the raw data or the existence of a formal option to request the raw data, and if references and validation for methodologies were provided.

Of the 281 papers (Supplement 2) retained for full reading, 24 were not retained for final analysis [review (6), no laboratory values presented (1), no access to full paper (8), duplicate (2), or not relevant to the topic (2), or other population(s) (former preterms, infants) (4), language (Arabic) (1)]. Ultimately, 257 papers were retained for data extraction. None of these papers used or referred to a publication standard to present neonatal laboratory data.

#### Countries and number of sites

Two hundred and fifty-four studies presented on the country of origin, with the United States (51), Turkey (26), India (22), Iran (14), Canada (12), South Korea (10), China (9), Germany (8), United Kingdom (8), and Egypt (7). Multinational papers were rare (3), and the study country was uncertain in another 3 papers. The majority were single-center studies (199), with significantly fewer (40) multicenter studies. For 7 papers, this information was not retrieved.

#### Number of neonates included and those with repeated measurements

In 250 papers, the median number of included neonates was 120 (range 10–66,526). If presented, 74/198 were single measurement dataset. In the papers (124) that clearly presented repeated measurements, the median number of neonates was 100 (range 2–26,871). Consequently, information on repeated measurements was unclear in a relevant portion (59/198).

#### Demographics

Using standard GA definitions, 154, 115, 117, and 125 papers reported data on term, preterm, very preterm or extreme preterm cases, respectively, with some information on subgroups in 225 papers. In 22 papers, the age characteristics were unclear. Data on sex were included in 210 papers, while information on the proportion of sex (male/female) was presented in 124 papers. The potential impact of sex was analyzed in only 6 papers. Clinically meaningful information on age (e.g., gestational or postnatal) at sampling was provided in 227/257 papers. Consequently, for a relevant portion (30/257) of papers, age-related aspects were limited to “neonate.” Whether neonates were outpatients, hospitalized, or both were included was mentioned in 32, 227, and 12 papers. At least some information on medical conditions (clinical information, medical diagnosis, or healthy) was presented in 236/257 papers. Information on drugs administered was only provided in 120/257 papers.

#### Laboratory values and analysis

Information on equipment was specified in 138/257 papers. For the analysis method, this was present in 135/257 of the papers. Bilirubin, hemogram, and sodium were the most commonly reported analytes.

Thresholds were applied as diagnostic criteria. For bilirubin, thresholds were commonly based on the Bhutani normogram (or similar), while there was relevant variability in thresholds for hyponatriemia (range 125–135 mmol/l) or hypernatriemia (range 145–150 mmol/l) or raised direct bilirubin (1–2 mg/dl). Point-of-care testing (excluding transcutaneous bilirubin measurement) was only involved in 2 papers. External laboratory use was involved in 24/257 papers.

#### Statistics

Statistics varied from basic descriptive, (para)metric analysis to Passing Bablok, Bland–Altman or Pearson correlations, multivariate, or general linear models. Statistics were assessed as informative and accurate in 242/257 papers. In contrast, validation efforts were only presented in 19/257 papers, the option to request for data access was present in 5 papers, and information on the references or validation of the analytical methods was retrieved in 66/257 papers. Information on reference interval estimation techniques was not extracted.

## Discussion

Based on this assessment of the literature, published information on laboratory values for neonates is sparse, not systematic, and incomplete. There is marked variation in how neonatal laboratory data, actionable thresholds, or reference ranges are presented. This variation undermines efforts to compare practices and establish quality standards for clinical care and research. No publication was identified that presented a publication standard for the planned INC work to develop laboratory reference ranges in neonates.^5^

As highlighted by Sun et al., inadequate reporting of analytical characteristics of biomarkers in clinical research is not unique to neonates, despite a statement by the Consortium of Laboratory Medicine Journal Editors to fully describe laboratory methods and specimen handling.^6,7^ However, neonates add further complexity, in part related to laboratory techniques (low volume samples, differences in plasma and blood composition) but mainly related to their biological variation (gestational and postnatal age).^2^

A next step should be a multidisciplinary effort to generate publication recommendations that present neonatal laboratory data, consistent with the existing approaches, but with considerations related to variability within the neonatal population. Such tools can subsequently be applied to the ongoing INC effort to define actionable reference ranges of commonly used laboratory values and will improve the impact of papers presenting laboratory values in neonates. This is critically important for investigators, industry, and regulators to facilitate multiple clinical trial processes, such as the development of inclusion and exclusion criteria, adverse event reporting, or outcome measures.

## Supplementary information

SUPPLEMENT 1 search string

Supplement 2 overview papers retained final

## Data Availability

The corresponding author can be contacted to share the raw data, if based on a reasonable request and study protocol.
