# Supplementary material for: The publication quality of laboratory values in clinical studies in neonates
Source: Pediatr Res. 2022 Dec 22;94(1):96–8. doi: 10.1038/s41390-022-02385-1 (PMC10356592; doi:10.1038/s41390-022-02385-1)
Supplement: Supplementary file 1 — SUPPLEMENT 1 search string [file 41390_2022_2385_MOESM1_ESM.pdf]

SUPPLEMENT 1

Search String: ((((((('blood collecting' OR 'blood collect*' OR 'blood sampl*' OR 'blood sampling' OR 'blood specimen collection' OR 'sampling, blood' OR 'function, kidney' OR 'kidney function' OR 'kidney physiology' OR 'renal function' OR 'fluid retention' OR 'fluid* retention' OR 'retention, fluid*' OR 'filtration fraction, kidney' OR 'glomerular filtration' OR 'glomerular filtration fraction' OR 'glomerular permeability' OR 'glomerulofiltration' OR 'glomerulus filtration' OR 'glomerulus hyperfiltration' OR 'glomerulus permeability' OR 'kidney filtration' OR 'kidney filtration fraction' OR 'kidney glomerular filtration' OR 'kidney glomerulus filtrate' OR 'kidney glomerulus filtration' OR 'kidney glomerulus permeability' OR 'renal filtration fraction' OR 'renal glomerular filtration' OR 'renal glomerulus filtration' OR 'kidney metabolism' OR 'metabolism, kidney' OR 'renal metabolism' OR 'kidney tubular function' OR 'kidney tubule function' OR 'kidney tubulus function' OR 'renal tubular function' OR 'renal tubule function' OR 'renal tubulus function' OR 'tubular function' OR 'acidification capacity, renal' OR 'kidney acidifying capacity' OR 'concentration mechanism kidney' OR 'kidney concentrating ability' OR 'kidney concentrating capacity' OR 'kidney concentrating mechanism' OR 'kidney concentration function' OR 'kidney concentration mechanism' OR 'kidney concentration test' OR 'renal concentrating ability' OR 'renal concentrating capacity' OR 'renal concentrating function' OR 'renal concentrating power' OR 'renal concentration capacity' OR 'urinary concentrating ability' OR 'urinary concentrating capacity' OR 'urinary concentration' OR 'urinary concentration capacity' OR 'urine concentrating ability' OR 'urine concentrating capacity' OR 'urine concentration' OR 'urine concentration capacity' OR 'urine concentration test' OR 'absorption, kidney tubule' OR 'kidney absorption' OR 'kidney reabsorption' OR 'kidney resorption' OR 'kidney sodium reabsorption' OR 'kidney sodium resorption' OR 'kidney transport' OR 'kidney tubular absorption' OR 'kidney tubular

reabsorption' OR 'kidney tubular transport' OR 'kidney tubule absorption' OR 'kidney tubule reabsorption' OR 'kidney tubule resorption' OR 'kidney tubule transport' OR 'kidney tubulus absorption' OR 'kidney tubulus reabsorption' OR 'kidney uptake' OR 'proximal tubule reabsorption' OR 'proximal tubule resorption' OR 'renal absorption' OR 'renal reabsorption' OR 'renal sodium reabsorption' OR 'renal transport' OR 'renal tubular absorption' OR 'renal tubular reabsorption' OR 'renal tubular resorption' OR 'renal tubular sodium reabsorption' OR 'renal tubular transport' OR 'renal tubule absorption' OR 'renal tubule reabsorption' OR 'renal tubulus absorption' OR 'tubular absorption' OR 'tubular reabsorption' OR 'tubular transport system' OR 'tubule absorption' OR 'tubule absorption, kidney' OR 'tubule reabsorption, kidney' OR 'tubulus reabsorption' OR 'sodium absorption' OR 'sodium reabsorption' OR 'apparent tubular excretion fraction' OR 'excretion, kidney tubule' OR 'kidney secretion' OR 'kidney tubular excretion' OR 'kidney tubule excretion' OR 'kidney tubule secretion' OR 'renal secretion' OR 'renal tubular excretion' OR 'renal tubular secretion' OR 'renal tubule excretion' OR 'tubular excretion' OR 'tubule excretion, kidney' OR 'kidney tubular potential' OR 'kidney tubule potential' OR 'potential, kidney tubule' OR 'renal tubular potential' OR 'renal tubule potential' OR 'tubule potential, kidney') NEAR/10 ('infant*' OR 'bab*' OR 'high risk infant*' OR 'hospitalised infant*' OR 'hospitalized infant*' OR 'child, newborn' OR 'full term infant*' OR 'human neonate*' OR 'newborn*' OR 'infant, newborn' OR 'neonate*' OR 'neonatus' OR 'newborn bab*' OR 'newborn child*' OR 'newborn infant*' OR 'newly born bab*' OR 'newly born child*' OR 'newly born infant*' OR 'extremely premature infant*' OR 'infant*, extremely premature' OR 'infant*, premature' OR 'infant*, premature, diseases' OR 'neonate*, premature' OR 'pre-mature infant*' OR 'pre-term bab*' OR 'pre-term child*' OR 'pre-term infant*' OR 'pre-term neonate*' OR 'pre-term newborn*' OR 'prematur*' OR 'premature bab*' OR 'premature birth' OR 'premature child*' OR 'premature childbirth' OR 'premature infant*'

OR 'premature infant disease*' OR 'premature infant diseases' OR 'premature neonate*' OR 'premature newborn*' OR 'premature syndrome*' OR 'prematuritas' OR 'prematurity' OR 'preterm bab*' OR 'preterm child*' OR 'preterm infant*' OR 'preterm neonate*' OR 'preterm newborn*') OR ('blood chemistry' OR 'blood chemistry' OR 'clinical chemistry' OR 'clinical chemistry' OR 'potassium' OR potassium OR 'bilirubin' OR bilirubin* OR 'creatinine' OR creatinine* OR 'hemoglobin' OR hemoglobin OR 'glucose blood level' OR 'glucose blood level*' OR 'alkaline phosphatase' OR 'alkaline phosphatase*' OR sodium OR 'alanine aminotransferase*' OR 'gamma glutamyltransferase*' OR 'blood analysis' OR 'blood analysis' OR 'blood identification' OR 'hematological examination' OR 'hematological examination' OR 'blood biochemistry' OR 'blood biochemical analysis' OR 'blood biochemistry' OR 'thrombography' OR 'thrombogram' OR 'thrombography' OR 'blood chemistry' OR 'blood chemical analysis' OR 'blood chemistry' OR 'blood gas analysis' OR 'blood gas analysis' OR 'blood gas measurement' OR 'blood gas test' OR 'transcutaneous carbon dioxide monitoring' OR 'co2 monitoring, transcutaneous' OR 'co2 tension monitoring, transcutaneous' OR 'carbon dioxide monitoring, transcutaneous' OR 'carbon dioxide tension monitoring, transcutaneous' OR 'monitoring, transcutaneous co2' OR 'monitoring, transcutaneous carbon dioxide' OR 'transcutaneous co2 measurement' OR 'transcutaneous co2 monitoring' OR 'transcutaneous carbon dioxide measurement' OR 'transcutaneous carbon dioxide monitoring' OR 'transcutaneous oxygen monitoring' OR 'blood gas monitoring, transcutaneous' OR 'oxygen tension monitoring, transcutaneous' OR 'transcutaneous blood gas monitoring' OR 'transcutaneous oxygen monitoring' OR 'transcutaneous oxygen tension' OR 'blood glucose monitoring' OR 'blood glucose control' OR 'blood glucose monitoring' OR 'blood glucose self-monitoring' OR 'monitoring, blood glucose' OR 'dried blood spot testing' OR 'dried blood spot' OR 'dried blood spot testing' OR 'dry blood spot' OR 'hemoglobin analysis' OR 'hemoglobin

analysis' OR 'hemoglobin analysis' OR 'hemoglobin determination' OR 'determination, hemoglobin' OR 'determination, hemoglobin' OR 'hemoglobin assay' OR 'hemoglobin concentration' OR 'hemoglobin determination' OR 'hemoglobin estimation' OR 'hemoglobinometry' OR 'hb determination' OR 'hemoglobin assay' OR 'hemoglobin concentration' OR 'hemoglobin determination' OR 'hemoglobin estimation' OR 'hemoglobinometry' OR 'petrosal sinus sampling' OR 'petrosal sinus sampling') NEAR/10 ('infant*' OR 'bab*' OR 'high risk infant*' OR 'hospitalised infant*' OR 'hospitalized infant*' OR 'child, newborn' OR 'full term infant*' OR 'human neonate*' OR 'newborn*' OR 'infant, newborn' OR 'neonate*' OR 'neonatus' OR 'newborn bab*' OR 'newborn child*' OR 'newborn infant*' OR 'newly born bab*' OR 'newly born child*' OR 'newly born infant*' OR 'extremely premature infant*' OR 'infant*, extremely premature' OR 'infant*, premature' OR 'infant*, premature, diseases' OR 'neonate*, premature' OR 'pre-mature infant*' OR 'pre-term bab*' OR 'pre-term child*' OR 'pre-term infant*' OR 'pre-term neonate*' OR 'pre-term newborn*' OR 'prematur*' OR 'premature bab*' OR 'premature birth' OR 'premature child*' OR 'premature childbirth' OR 'premature infant*' OR 'premature infant disease*' OR 'premature infant diseases' OR 'premature neonate*' OR 'premature newborn*' OR 'premature syndrome*' OR 'prematuritas' OR 'prematurity' OR 'preterm bab*' OR 'preterm child*' OR 'preterm infant*' OR 'preterm neonate*' OR 'preterm newborn*')) AND ('article'/it OR 'article in press'/it OR 'review'/it) AND ('case control study'/de OR 'clinical protocol'/de OR 'clinical study'/de OR 'clinical trial'/de OR 'clinical trial topic'/de OR 'cohort analysis'/de OR 'comparative effectiveness'/de OR 'comparative study'/de OR 'controlled clinical trial'/de OR 'controlled clinical trial topic'/de OR 'controlled study'/de OR 'correlational study'/de OR 'cross sectional study'/de OR 'crossover procedure'/de OR 'data collection method'/de OR 'diagnostic test accuracy study'/de OR 'double blind procedure'/de OR 'evidence based

medicine'/de OR 'evidence based practice'/de OR 'feasibility study'/de OR 'field study'/de OR 'human experiment'/de OR 'intermethod comparison'/de OR 'longitudinal study'/de OR 'major clinical study'/de OR 'meta analysis'/de OR 'meta analysis topic'/de OR 'methodology'/de OR 'multicenter study'/de OR 'multicenter study topic'/de OR 'observational study'/de OR 'open study'/de OR 'phase 1 clinical trial'/de OR 'phase 1 clinical trial topic'/de OR 'phase 2 clinical trial'/de OR 'phase 2 clinical trial topic'/de OR 'phase 3 clinical trial'/de OR 'phase 4 clinical trial'/de OR 'phase 4 clinical trial topic'/de OR 'pilot study'/de OR 'population based case control study'/de OR 'prospective study'/de OR 'quality control'/de OR 'randomized controlled trial'/de OR 'randomized controlled trial topic'/de OR 'retrospective study'/de OR 'statistical model'/de OR 'systematic review'/de OR 'systematic review topic'/de OR 'theoretical model'/de OR 'validation process'/de OR 'validation study'/de)) AND [humans]/lim AND [1989-2021]/py) AND [english]/lim) AND [2000-2021]/py) AND ([infant]/lim OR [newborn]/lim)) AND (('hemoglobin'/exp OR 'ferrohemoglobin':ti,ab OR 'ferrohemoglobin':ti,ab OR 'free hemoglobin':ti,ab OR 'free hemoglobin':ti,ab OR 'hemoglobin':ti,ab OR 'hemoglobine':ti,ab OR 'hemoglobins':ti,ab OR 'hemoglobin':ti,ab OR 'hemoglobine':ti,ab OR 'hemoglobins':ti,ab OR 'hemoglobulin':ti,ab OR 'trm 645':ti,ab OR 'trm645':ti,ab OR 'unstable hemoglobin':ti,ab OR 'unstable hemoglobin':ti,ab) OR ('erythrocyte'/exp OR 'rbc':ti,ab OR 'erythrocyte':ti,ab OR 'erythrocyte concentration':ti,ab OR 'erythrocyte incorporation':ti,ab OR 'erythrocyte population':ti,ab OR 'erythrocyte stroma':ti,ab OR 'erythrocyte typing':ti,ab OR 'erythrocytes':ti,ab OR 'erythrocytic cell':ti,ab OR 'red blood cell':ti,ab OR 'red blood cell stroma':ti,ab OR 'red blood corpuscle':ti,ab OR 'red cell':ti,ab OR 'red cell stroma':ti,ab OR 'stroma, erythrocyte':ti,ab OR 'washed erythrocyte':ti,ab) OR ('leukocyte'/exp OR 'human leucocyte':ti,ab OR 'human leukocyte':ti,ab OR 'leucocyte':ti,ab OR 'leucocytes':ti,ab OR 'leukocyte':ti,ab OR 'leukocytes':ti,ab OR

'peripheral blood leucocyte':ti,ab OR 'peripheral blood leukocyte':ti,ab OR 'peripheral leucocyte':ti,ab OR 'peripheral leukocyte':ti,ab OR 'wbc':ti,ab OR 'white blood cell':ti,ab OR 'white blood corpuscle':ti,ab OR 'white cell':ti,ab) OR ('thrombocyte'/exp OR 'blood platelet':ti,ab OR 'blood platelets':ti,ab OR 'platelet':ti,ab OR 'thrombocyte':ti,ab) OR ('sodium'/exp OR 'na':ti,ab OR 'natrium':ti,ab OR 'sodium':ti,ab OR 'sodium isotopes':ti,ab OR 'sodium radioisotopes':ti,ab OR 'sodium salt':ti,ab OR 'sodium space':ti,ab OR 'toxynan':ti,ab) OR ('potassium'/exp OR 'k 39':ti,ab OR 'kalium':ti,ab OR 'potassium':ti,ab OR 'potassium content':ti,ab OR 'potassium isotopes':ti,ab OR 'potassium radioisotopes':ti,ab) OR ('calcium'/exp OR '40ca':ti,ab OR 'ca':ti,ab OR 'calcium':ti,ab OR 'calcium 40':ti,ab OR 'calcium content':ti,ab OR 'calcium deposition':ti,ab OR 'calcium isotopes':ti,ab OR 'calcium radioisotopes':ti,ab OR 'phosphorus'/exp OR 'fosfor':ti,ab OR 'phosphor':ti,ab OR 'phosphorus':ti,ab OR 'phosphorus isotopes':ti,ab OR 'phosphorus radioisotopes':ti,ab OR 'phosphorus, inorganic':ti,ab OR 'phosphorus, radioactive':ti,ab OR 'radio phosphorus':ti,ab OR 'radioactive phosphorus':ti,ab OR 'radiophosphorus':ti,ab OR 'white phosphorus':ti,ab OR 'yellow phosphorus':ti,ab OR 'glucose'/exp OR 'cartose':ti,ab OR 'corn sugar':ti,ab OR 'd glucose':ti,ab OR 'dextro glucose':ti,ab OR 'dextropur':ti,ab OR 'dextrose':ti,ab OR 'dextrose 10%':ti,ab OR 'dextrose 2.5%':ti,ab OR 'dextrose 20%':ti,ab OR 'dextrose 25%':ti,ab OR 'dextrose 30%':ti,ab OR 'dextrose 38.5%':ti,ab OR 'dextrose 40%':ti,ab OR 'dextrose 5%':ti,ab OR 'dextrose 50%':ti,ab OR 'dextrose 60%':ti,ab OR 'dextrose 7.7%':ti,ab OR 'dextrose 70%':ti,ab OR 'dextrosol':ti,ab OR 'glucodin':ti,ab OR 'glucola':ti,ab OR 'glucolin':ti,ab OR 'glucose':ti,ab OR 'glucose hypotonic solution':ti,ab OR 'glucose influx':ti,ab OR 'glucose medium':ti,ab OR 'glucose solution':ti,ab OR 'glucose solution, hypertonic':ti,ab OR 'glutol':ti,ab OR 'glycose':ti,ab OR 'glycovarin':ti,ab OR 'grape sugar':ti,ab OR 'hypertonic dextrose solution':ti,ab OR 'hypertonic glucose solution':ti,ab OR 'hypotonic glucose':ti,ab OR

'hypotonic glucose solution':ti,ab OR 'koladex':ti,ab OR 'saccharum amylaceum':ti,ab OR 'starch sugar':ti,ab OR 'vamin glucose':ti,ab OR 'creatinine'/exp OR '1 methylglycocyamidine':ti,ab OR '1 methylhydantoin 1 imide':ti,ab OR '2 imino 1 methyl 4 imidazolinone':ti,ab OR 'creatinin':ti,ab OR 'creatinine':ti,ab OR 'creatinine hydrochloride':ti,ab OR 'kreatinine':ti,ab OR 'methylglycocyamimine':ti,ab OR 'urea nitrogen blood level'/exp OR 'bun':ti,ab OR 'blood urea nitrogen':ti,ab OR 'plasma urea nitrogen':ti,ab OR 'serum bun':ti,ab OR 'serum urea nitrogen':ti,ab OR 'urea nitrogen blood level':ti,ab OR 'bilirubin'/exp OR '1, 3, 6, 7 tetramethyl 4, 5 dicarboxyethyl 2, 8 divinyl (b 13) dihydrobilenone':ti,ab OR 'bilirubin':ti,ab OR 'bilirubin acid':ti,ab OR 'bilirubin delta':ti,ab OR 'bilirubin ix alpha':ti,ab OR 'bilirubin ixalpha':ti,ab OR 'bilirubin pigment':ti,ab OR 'bilirubin sulfate isomer':ti,ab OR 'bilirubin sulphate isomer':ti,ab OR 'bilirubinate':ti,ab OR 'bilirubine':ti,ab OR 'bilirubinoid':ti,ab OR 'calcium bilirubinate':ti,ab OR 'hematoidin':ti,ab OR 'hematoidin':ti,ab OR 'indirect bilirubin':ti,ab OR 'mesobilirubin':ti,ab OR 'unconjugated bilirubin':ti,ab OR 'aspartate aminotransferase'/exp OR '1 aspartate 2 oxoglutarate aminotransferase':ti,ab OR '1 aspartate:2 oxoglutarate aminotransferase':ti,ab OR 'got':ti,ab OR 'aminotransferase, aspartate':ti,ab OR 'aspartate amino transferase':ti,ab OR 'aspartate aminotransferase':ti,ab OR 'aspartate aminotransferases':ti,ab OR 'aspartate transaminase':ti,ab OR 'aspartic aminotransferase':ti,ab OR 'aspartic transaminase':ti,ab OR 'e.c. 2.6.1.1':ti,ab OR 'glutamate aspartate transaminase':ti,ab OR 'glutamate oxalacetate transaminase':ti,ab OR 'glutamate oxalacetic transaminase':ti,ab OR 'glutamate oxalate transaminase':ti,ab OR 'glutamate oxalic transaminase':ti,ab OR 'glutamate oxaloacetate aminotransferase':ti,ab OR 'glutamate oxaloacetate transaminase':ti,ab OR 'glutamate oxaloacetic acid transaminase':ti,ab OR 'glutamatoxalacetate transaminase':ti,ab OR 'glutamic aspartic aminotransferase':ti,ab OR 'glutamic aspartic transaminase':ti,ab OR 'glutamic oxal

acetatic transaminase':ti,ab OR 'glutamic oxalacetic acid transaminase':ti,ab OR 'glutamic oxalacetic transaminase':ti,ab OR 'glutamic oxalacetic transferase':ti,ab OR 'glutamic oxalic transaminase':ti,ab OR 'glutamic oxaloacetic acid transaminase':ti,ab OR 'glutamic oxaloacetic aminotransferase':ti,ab OR 'glutamic oxaloacetic transaminase':ti,ab OR 'glutamine oxaloacetic transaminase':ti,ab OR 'glutaminic oxalacetic transaminase':ti,ab OR 'l asparate 2 oxoglutarate transaminase':ti,ab OR 'l aspartate 2 oxoglutarate aminotransferase':ti,ab OR 'l aspartate aminotransferase':ti,ab OR 'l aspartate:2 oxoglutarate aminotransferase':ti,ab OR 'l aspartate:2 oxoglutarate transaminase':ti,ab OR 'l aspatate:2 oxoglutarate aminotransferase':ti,ab OR 'levo aspartate aminotransferase':ti,ab OR 'transaminase a':ti,ab OR 'alanine aminotransferase'/exp OR 'gpt':ti,ab OR 'alanin aminotransferase':ti,ab OR 'alanine 2 oxoglutarate aminotransferase':ti,ab OR 'alanine 2 oxoisovalerate aminotransferase':ti,ab OR 'alanine alpha ketoglutarate transaminase':ti,ab OR 'alanine alpha oxoglutarate transaminase':ti,ab OR 'alanine amino transferase':ti,ab OR 'alanine aminotransferase':ti,ab OR 'alanine transaminase':ti,ab OR 'alanine transpeptidase':ti,ab OR 'alanine:2 oxoglutarate aminotransferase':ti,ab OR 'e.c. 2.6.1.2':ti,ab OR 'glutamate alanine transaminase':ti,ab OR 'glutamate pyruvate aminotransferase':ti,ab OR 'glutamate pyruvate transaminase':ti,ab OR 'glutamate pyruvatetransaminase':ti,ab OR 'glutamic alanine aminotransferase':ti,ab OR 'glutamic pyruvate transaminase':ti,ab OR 'glutamic pyruvic aminotransferase':ti,ab OR 'glutamic pyruvic transaminase':ti,ab OR 'glutamopyruvic transaminase':ti,ab OR 'l alanine 2 oxoglutarate aminotransferase':ti,ab OR 'l alanine:2 oxoglutarate aminotransferase':ti,ab OR 'alkaline phosphatase'/exp OR 'alcalic phosphatase':ti,ab OR 'alkali phosphatase':ti,ab OR 'alkalic phosphatase':ti,ab OR 'alkaline monophosphoesterase':ti,ab OR 'alkaline phosphatase':ti,ab OR 'alkaline phosphohydrolase':ti,ab OR 'alkaline phosphomonoesterase':ti,ab OR 'alkalinic

phosphatase':ti,ab OR 'basic phosphatase':ti,ab OR 'e.c. 3.1.3.1':ti,ab OR 'heat stable alkaline phosphatase':ti,ab OR 'orthophosphoric monoester phosphohydrolase':ti,ab OR 'phosphatase, alkaline':ti,ab OR 'c reactive protein'/exp OR 'c reactive protein':ti,ab OR 'c reaction protein':ti,ab OR 'c-reactive protein':ti,ab OR 'creactive protein':ti,ab OR 'crp':ti,ab OR 'protein, c reactive':ti,ab OR 'serum c reactive protein':ti,ab OR 'procalcitonin'/exp OR 'calcitonin precursor':ti,ab OR 'procalcitonin':ti,ab)
