# Supplementary material for: The publication quality of laboratory values in clinical studies in neonates
Source: Pediatr Res. 2022 Dec 22;94(1):96–8. doi: 10.1038/s41390-022-02385-1 (PMC10356592; doi:10.1038/s41390-022-02385-1)
Supplement: Supplementary file 2 — Supplement 2 overview papers retained final [file 41390_2022_2385_MOESM2_ESM.pdf]

Full Text Link/embase	Title
http://dx.doi.org/10.1542/peds.2005-0590	Bilirubin measurement for neonates: Comparison of 9 frequently used methods
http://dx.doi.org/10.1111/ped.14251	Hour-specific nomogram for transcutaneous Bilirubin in newborns in Myanmar
http://dx.doi.org/10.1080/14767058.2017.1390561	Accuracy of transcutaneous bilirubinometry in the preterm infants: a comprehensive meta-analysis
http://dx.doi.org/10.17219/acem/110328	EGFR values and selected renal urine biomarkers in preterm neonates with uncomplicated clinical course
http://dx.doi.org/10.1371/journal.pone.0084892	Critical serum creatinine values in very preterm newborns
http://dx.doi.org/10.1111/ped.12149	Hour-specific nomogram for transcutaneous bilirubin in Japanese neonates
http://dx.doi.org/10.1038/s41390-020-0977-4	Creatinine filtration kinetics in critically Ill neonates
http://dx.doi.org/10.1155/2013/316430	Factors affecting bilirubin levels during first 48 hours of life in healthy infants
http://dx.doi.org/10.1542/peds.2006-3024	Ontogeny of bilirubin-binding capacity and the effect of clinical status in premature infants born at less than 1300 grams
http://dx.doi.org/10.3945/jn.113.177915	Changes in soluble transferrin receptor and hemoglobin concentrations inmalawianmothers are associated with those values in their exclusively breastfed, HIV-exposed infants
http://dx.doi.org/10.1038/sj.jp.7211905	A gestation- and postnatal age-based reference chart for assessing renal function in extremely premature infants
https://www.embase.com/search/results?subaction=viewrecord&id=L2002159290&from=export	Cord, venous and capillary hemoglobin values in healthy neonates
http://dx.doi.org/10.1002/14651858.CD012660	Transcutaneous bilirubinometry versus total serum bilirubin measurement for newborns
http://dx.doi.org/10.1007/s13312-017-1108-0	Transcutaneous bilirubin nomogram for healthy term and late preterm neonates in first 96 hours of life
http://dx.doi.org/10.1007/s00467-016-3427-0	Normal values of urine total protein- and albumin-to-creatinine ratios in term newborns
http://dx.doi.org/10.1136/adc.82.1.71	Reference ranges for plasma cystatin C and creatinine measurements in premature infants, neonates, and older children
http://dx.doi.org/10.1055/s-0041-1724000	Gestational and Age-Specific Cystatin C Reference Intervals for Newborns
http://dx.doi.org/10.1001/archpediatrics.2010.242	Hour-specific bilirubin nomogram in infants with ABO incompatibility and direct coombs-positive results
http://dx.doi.org/10.1016/j.clinbiochem.2011.06.987	Preliminary reference intervals for cystatin C and beta-trace protein in preterm and term neonates
http://dx.doi.org/10.1111/j.1651-2227.2007.00622.x	Skin bilirubin nomogram for the first 96 h of life in a European normal healthy newborn population, obtained with multiwavelength transcutaneous bilirubinometry
http://dx.doi.org/10.1007/s00467-008-0867-1	Determination of reference values for plasma cystatin C and comparison with creatinine in premature infants
http://dx.doi.org/10.1007/s00467-020-04838-3	Distribution of proteinuria- and albuminuria-to-creatinine ratios in preterm newborns
http://dx.doi.org/10.1016/j.clinbiochem.2014.07.012	Redefining normal bone and mineral clinical biochemistry reference intervals for healthy infants in Canada
http://dx.doi.org/10.1016/j.jpeds.2014.02.029	Glucose-6-phosphate dehydrogenase activity levels in white newborn infants
http://dx.doi.org/10.1542/peds.2008-2654	Reference ranges for hematocrit and blood hemoglobin concentration during the neonatal period: Data from a multihospital health care system
http://dx.doi.org/10.3109/14767058.2012.657277	Creatinine reference values in ELBW infants: Impact of quantification by Jaffe or enzymatic method
http://dx.doi.org/10.1016/j.jpeds.2010.04.076	Transcutaneous bilirubin levels in late preterm neonates
http://dx.doi.org/10.1007/s12519-014-0468-y	Hemoglobin cut-off values in healthy Turkish infants
http://dx.doi.org/10.1080/03630260600867883	Accurate measurements of fetal hemoglobin for neonates with different gestational ages
https://www.embase.com/search/results?subaction=viewrecord&id=L354714454&from=export	Reference values for urinary calcium, sodium and potassium in healthy newborns, infants and children

http://dx.doi.org/10.1111/j.1365-2257.2006.00767.x	Complete blood count parameters for healthy, small-for-gestational-age, full-term newborns
http://dx.doi.org/10.4103/2249-4847.199758	Hematological reference values for full-term, healthy newborns of Jeddah, Saudi Arabia
http://dx.doi.org/10.1007/s00467-013-2632-3	Reference intervals of serum cystatin C/creatinine ratio of 30 postnatal days in neonates
http://dx.doi.org/10.1007/s004670000356	Progression of renal function in preterm neonates with gestational age ≤32 weeks
http://dx.doi.org/10.1038/jp.2010.82	Elevation in plasma creatinine and renal failure in premature neonates without major anomalies: Terminology, occurrence and factors associated with increased risk
http://dx.doi.org/10.1136/adc.2008.152454	Plasma aminotransferase concentrations in preterm infants
http://dx.doi.org/10.1186/1471-2431-9-49	Selected hematologic and biochemical measurements in African HIV-infected and uninfected pregnant women and their infants: The HIV prevention trials network 024 protocol
http://dx.doi.org/10.1016/j.jpeds.2017.07.035	Alkaline Phosphatase in Infant Cardiopulmonary Bypass: Kinetics and Relationship to Organ Injury and Major Cardiovascular Events
http://dx.doi.org/10.1136/adc.2006.105361	Prospective surveillance study of severe hyperbilirubinaemia in the newborn in the UK and Ireland
http://dx.doi.org/10.1016/j.jpeds.2010.05.024	Hemolysis and hyperbilirubinemia in antiglobulin positive, direct ABO blood group heterospecific neonates
http://dx.doi.org/10.1007/s00431-014-2373-8	Intermittent versus continuous phototherapy for the treatment of neonatal non-hemolytic moderate hyperbilirubinemia in infants more than 34 weeks of gestational age: a randomized controlled trial
http://dx.doi.org/10.3109/14767058.2012.733767	Renal function is impaired in small for gestational age premature infants
http://dx.doi.org/10.1038/jp.2016.259	Utility of measuring direct bilirubin at first 72 h of age in neonates admitted to the neonatal intensive care unit
http://dx.doi.org/10.1186/s12887-020-02260-0	Mutation spectrum and biochemical features in infants with neonatal Dubin-Johnson syndrome
http://dx.doi.org/10.1007/s00467-016-3572-5	Increased urinary neutrophil gelatinase-associated lipocalin in very-low-birth-weight infants with oliguria and normal serum creatinine
http://dx.doi.org/10.1016/j.earlhumdev.2016.04.008	Influence of hyperbilirubinemia on neonatal sucking
http://dx.doi.org/10.1203/00006450-200112000-00012	Are moderate degrees of hyperbilirubinemia in healthy term neonates really safe for the brain?
http://dx.doi.org/10.3233/NPM-200511	Neurodevelopmental outcome of healthy term newborn with serum bilirubin >15 mg/dl at one year
https://www.embase.com/search/results?subaction=viewrecord&id=L358409105&from=export	Neonatal jaundice - Are we over-treating?
http://dx.doi.org/10.1177/2150135118775413	Acute Kidney Injury Defined by Fluid Corrected Creatinine in Neonates After the Norwood Procedure
http://dx.doi.org/10.1177/0148607115626921	Preventing the Progression of Intestinal Failure-Associated Liver Disease in Infants Using a Composite Lipid Emulsion: A Pilot Randomized Controlled Trial of SMOFlipid
http://dx.doi.org/10.1093/jpids/pis036	Laboratory abnormalities among HIV-exposed, uninfected infants: IMPAACT protocol P1025
http://dx.doi.org/10.1055/s-0029-1243315	Treatment of patent ductus arteriosus: Indomethacin or ibuprofen?
http://dx.doi.org/10.1203/PDR.0b013e3181ac4a30	Breast milk jaundice correlates with high levels of epidermal growth factor
http://dx.doi.org/10.1007/s12098-013-1068-x	Evaluation of renal function in term babies with perinatal asphyxia
https://www.embase.com/search/results?subaction=viewrecord&id=L365377696&from=export	Utility of measuring direct bilirubin at 12-24 hours of age in neonates admitted to the neonatal intensive care unit
http://dx.doi.org/10.1016/j.jpurol.2020.06.030	Kidney injury molecule-1/creatinine as a urinary biomarker of acute kidney injury in critically ill neonates
http://dx.doi.org/10.1371/journal.pone.0062858	Evaluation of Treatment Thresholds for Unconjugated Hyperbilirubinemia in Preterm Infants: Effects on Serum Bilirubin and on Hearing Loss?
https://www.embase.com/search/results?subaction=viewrecord&id=L369882569&from=export	Is elevated mean corpuscular hemoglobin concentration valuable for neonatal hereditary spherocytosis screening?
http://dx.doi.org/10.1542/peds.113.4.775	Incidence, Course, and Prediction of Hyperbilirubinemia in Near-Term and Term Newborns
https://www.embase.com/search/results?subaction=viewrecord&id=L361141906&from=export	Neonatal hypernatremia and dehydration in infants receiving inadequate breastfeeding

http://dx.doi.org/10.1016/j.earlhumdev.2011.07.001	Validation of transcutaneous bilirubin nomogram in identifying neonates not at risk of hyperbilirubinaemia: A prospective, observational, multicenter study
http://dx.doi.org/10.1177/0300060520977442	Value of urinary kidney injury molecule-1 levels in predicting acute kidney injury in very low birth weight preterm infants
http://dx.doi.org/10.1093/tropej/fml067	The frequency of UDP-glucuronosyltransferase 1 A1 promoter region (TA)7 polymorphism in newborns and it's relation with jaundice
https://www.embase.com/search/results?subaction=viewrecord&id=L369526602&from=export	Early predictors of pathological jaundice due to ABO hemolytic disease
https://www.embase.com/search/results?subaction=viewrecord&id=L358423629&from=export	The effect of clofibrate on decreasing serum bilirubin in healthy term neonates under home phototherapy
http://dx.doi.org/10.1016/j.clinbiochem.2008.09.108	Clinical implication of the difference between transcutaneous bilirubinometry and total serum bilirubin for the classification of newborns at risk of hyperbilirubinemia
http://dx.doi.org/10.1542/peds.2016-4009	Chronic auditory toxicity in late preterm and term infants with significant hyperbilirubinemia
http://dx.doi.org/10.1007/s12098-011-0394-0	Fluid supplementation in hyperbilirubinemia
http://dx.doi.org/10.1007/s00431-004-1533-7	Detection of hyperbilirubinaemia in jaundiced full-term neonates by eye or by bilirubinometer?
https://www.embase.com/search/results?subaction=viewrecord&id=L43349487&from=export	Urinary tract infection and hyperbilirubinemia
http://dx.doi.org/10.1038/s41372-019-0359-0	Dysnatremia in extremely low birth weight infants is associated with multiple adverse outcomes
http://dx.doi.org/10.22038/ijn.2017.20577.1231	Risk factors and neurological outcomes of neonatal hypernatremia
http://dx.doi.org/10.1159/000285571	Maturation of adrenal function determined by urinary glucocorticoid steroid excretion rates in preterm infants of more than 30 weeks of gestational age
http://dx.doi.org/10.3923/ijp.2006.455.458	Effect of cotoneaster tricolor pojark manna on serum bilirubin levels in neonates
http://dx.doi.org/10.1590/s1808-86942009000300013	Analysis of transient otoacoustic emissions and brainstem evoked auditory potentials in neonates with hyperbilirubinemia
http://dx.doi.org/10.1016/j.athoracsur.2016.05.049	Outcomes Using a Conservative Versus Liberal Red Blood Cell Transfusion Strategy in Infants Requiring Cardiac Operation
http://dx.doi.org/10.1038/s41390-019-0415-7	Rate of rise of total serum bilirubin in very low birth weight preterm infants
http://dx.doi.org/10.1038/jp.2013.140	UGT1A1 gene variants and clinical risk factors modulate hyperbilirubinemia risk in newborns
https://www.embase.com/search/results?subaction=viewrecord&id=L2006866303&from=export	Comparison of the effect of phototherapy with oral calcium versus phototherapy alone in the treatment of unconjugated hyperbilirubinemia in healthy term infants
https://www.embase.com/search/results?subaction=viewrecord&id=L47248848&from=export	The early use of recombinant human erythropoietin in anemia of prematurity
https://www.embase.com/search/results?subaction=viewrecord&id=L351212121&from=export	The prognostic value of serum gamma glutamyltransferase activity in Chinese infants with previously diagnosed idiopathic neonatal hepatitis
http://dx.doi.org/10.7860/JCDR/2018/31791.11066	Weight loss and/or hypernatraemia in inadequately breastfed term neonates having non-haemolytic unconjugated hyperbilirubinaemia
http://dx.doi.org/10.1007/s00431-008-0841-8	Prevention of hypernatraemic dehydration in breastfed newborn infants by daily weighing
http://dx.doi.org/10.1007/s12519-013-0433-1	Urinary tract infections in neonates with jaundice in their first two weeks of life
http://dx.doi.org/10.1007/s12519-018-0119-9	Incidence and risk factors of post-phototherapy neonatal rebound hyperbilirubinemia
http://dx.doi.org/10.1007/s00134-004-2309-2	Does red blood cell transfusion change the near infra red photoplethysmography signal in infants?
http://dx.doi.org/10.1002/jpen.2004	Soybean-Oil Lipid Minimization for Prevention of Intestinal Failure–Associated Liver Disease in Late-Preterm and Term Infants With Gastrointestinal Surgical Disorders
http://dx.doi.org/10.1016/j.braindev.2015.01.001	Serum unbound bilirubin as a predictor for clinical kernicterus in extremely low birth weight infants at a late age in the neonatal intensive care unit
http://dx.doi.org/10.18621/eurj.373386	The relationship between hypernatremia and breast milk sodium levels in newborns with hypernatremic dehydration
http://dx.doi.org/10.4103/1319-2442.215148	Serum cystatin C as an earlier predictor of acute kidney injury than serum creatinine in preterm neonates with respiratory distress syndrome
http://dx.doi.org/10.1345/aph.1Q292	Safety and efficacy of two potassium cocktail formulations for treatment of neonatal hyperkalemia

http://dx.doi.org/10.18869/acadpub.ibj.21.3.182	An increased genotoxic risk in lymphocytes from phototherapy-treated hyperbilirubinemic neonates
http://dx.doi.org/10.1038/jp.2010.137	Acute neurological findings in a national cohort of neonates with severe neonatal hyperbilirubinemia
https://www.embase.com/search/results?subaction=viewrecord&id=L2002837088&from=export	Frequency of acute kidney injury in neonates admitted to Nicu at Shifa international hospital Islamabad
http://dx.doi.org/10.1016/j.pedneo.2012.08.011	Oral ibuprofen versus intravenous indomethacin for closure of patent ductus arteriosus in very low birth weight infants
http://dx.doi.org/10.1016/j.jpurol.2019.12.001	Plasma cystatin C versus renal resistive index as early predictors of acute kidney injury in critically ill neonates
http://dx.doi.org/10.1055/s-0040-1716845	The Impact of Hypernatremia in Preterm Infants on Neurodevelopmental Outcome at 18 Months of Corrected Age
http://dx.doi.org/10.1007/s12272-012-0919-8	Comparative evaluation for the use of oral ibuprofen and intravenous indomethacin in Korean infants with patent ductus
http://dx.doi.org/10.1002/jcla.21516	The Clinical Significance of Serum Cystatin Cin Critically Ill Newborns With Normal Serum Creatinine
https://www.embase.com/search/results?subaction=viewrecord&id=L38525332&from=export	Efficacy of Oral Phenobarbitone in Term "At Risk" Neonates in Decreasing Neonatal Hyperbilirubinemia: A Randomized Double-blinded, Placebo Controlled Trial
http://dx.doi.org/10.1016/j.jpeds.2012.02.003	Randomized controlled trial of early parenteral nutrition cycling to prevent cholestasis in very low birth weight infants
https://www.embase.com/search/results?subaction=viewrecord&id=L605180933&from=export	Analysis of factors affecting the prognosis of neonatal cholestasis
http://dx.doi.org/10.1007/s00467-014-2837-0	Acute kidney injury in preterm infants admitted to a neonatal intensive care unit
http://dx.doi.org/10.1258/096914103769010996	Population screening for neonatal liver disease: Potential for a community-based programme
http://dx.doi.org/10.3329/bjms.v13i1.17430	Evaluation of attributes to hyperbilirubinaemia in neonates in a tertiary care hospital in the Dhaka city
https://www.embase.com/search/results?subaction=viewrecord&id=L38744505&from=export	Once versus twice daily dose of gentamicin therapy in Thai neonates
http://dx.doi.org/10.1177/0148607107031006487	Effects of protein/nonprotein caloric intake on parenteral nutrition-associated cholestasis in premature infants weighing 600-1000 grams
http://dx.doi.org/10.1136/fn.83.2.f117	Blood glucose levels in a population of healthy, breast fed, term infants of appropriate size for gestational age
http://dx.doi.org/10.1016/j.pedneo.2018.07.005	Early predictors of neonatal hyperbilirubinemia in full term newborn
http://dx.doi.org/10.1038/pr.2016.70	Early urinary biomarkers of acute kidney injury in preterm infants
http://dx.doi.org/10.1002/jpen.1426	Early Hypophosphatemia in High-Risk Preterm Infants: Efficacy and Safety of Sodium Glycerophosphate From First Day on Parenteral Nutrition
http://dx.doi.org/10.1080/14767058.2020.1818216	Reticulocyte hemoglobin concentration for screening iron deficiency in very low birth weight preterm neonates
http://dx.doi.org/10.1016/j.jpeds.2006.02.011	Neonatal hyperbilirubinemia in African American males: The importance of glucose-6-phosphate dehydrogenase deficiency
http://dx.doi.org/10.1159/000490369	Perinatal Anemia is Associated with Neonatal and Neurodevelopmental Outcomes in Infants with Moderate to Severe Perinatal Asphyxia
http://dx.doi.org/10.1111/apa.13093	Extremely preterm infants who are small for gestational age have a high risk of early hypophosphatemia and hypokalemia
http://dx.doi.org/10.1007/s00467-003-1402-z	Indomethacin and renal impairment in neonates
http://dx.doi.org/10.1055/s-0041-1725119	Anemia after Pediatric Congenital Heart Surgery
http://dx.doi.org/10.5812/ijp.82103	Early phototherapy at lower total serum bilirubin can decrease auditory neuropathy disorder
http://dx.doi.org/10.1093/tropej/fmt023	Risk factors associated with unconjugated neonatal hyperbilirubinemia in Malaysian Neonates
https://www.embase.com/search/results?subaction=viewrecord&id=L35586219&from=export	Nutritional factors that affect the postnatal metabolic adaptation of full-term small- and large-for-gestational-age infants.
http://dx.doi.org/10.1002/jpen.2011	Incidence of Complications Associated With Parenteral Nutrition in Preterm Infants < 32 Weeks With a Mixed Oil Lipid Emulsion vs a Soybean Oil Lipid Emulsion in a Level IV Neonatal Intensive Care Unit
http://dx.doi.org/10.1177/0884533616687532	Retrospective Dual-Center Study of Parenteral Nutrition–Associated Cholestasis in Premature Neonates: 15 Years' Experience

http://dx.doi.org/10.1007/s12098-009-0335-3	Predicting neonatal hyperbilirubinemia using first day serum bilirubin levels
http://dx.doi.org/10.1080/080352501316978084	Manganese intake and cholestatic jaundice in neonates receiving parenteral nutrition: A randomized controlled study
http://dx.doi.org/10.1136/gut.48.3.409	The causes of obvious jaundice in South West Wales: Perceptions versus reality
http://dx.doi.org/10.1177/088453361668753	Retrospective dual-center study of parenteral nutrition-associated cholestasis in premature neonates: 15 years' experience
http://dx.doi.org/10.1177/00912700122010898	The influences of renal function and maturation on vancomycin elimination in newborns and infants
https://www.embase.com/search/results?subaction=viewrecord&id=L365695174&from=export	Cord blood bilirubin level as an early predictor of neonatal hyperbilirubinemia - A hospital-based prospective study
http://dx.doi.org/10.1111/j.1440-1754.2005.00688.x	Weight loss and hypernatremia in breast-fed babies: Frequency in neonates with non-hemolytic jaundice
http://dx.doi.org/10.3233/NPM-18138	Enteral fish oil supplementation in the resolution of parenteral nutrition associated cholestasis
http://dx.doi.org/10.1038/s41372-021-00995-x	Prophylactic Indomethacin in extremely preterm infants: association with death or BPD and observed early serum creatinine levels
http://dx.doi.org/10.3126/jnps.v34i1.9030	Adverse events of exchange transfusion in neonatal hyperbilirubinemia
http://dx.doi.org/10.5812/ijp.65742	Neonatal hypernatremic dehydration and thrombocytopenia: Its prevalence and relationship with prognosis
http://dx.doi.org/10.1007/s12098-011-0501-2	Association between peak serum bilirubin and neurodevelopmental outcomes in term babies with hyperbilirubinemia
https://www.embase.com/search/results?subaction=viewrecord&id=L39116202&from=export	Effects of fluid and electrolyte management on amphotericin B-induced nephrotoxicity among extremely low birth weight infants.
http://dx.doi.org/10.1038/srep22717	Association between anemia and bronchopulmonary dysplasia in preterm infants
http://dx.doi.org/10.1093/tropej/fmz051	Is routine monitoring for hypoglycemia required in intramural asymptomatic infant of diabetic mother? An audit in a tertiary care hospital
http://dx.doi.org/10.1111/pai.12607	Elevated blood eosinophils in early infancy are predictive of atopic dermatitis in children with risk for atopy
http://dx.doi.org/10.1081/JDI-200026749	Acute renal failure in the neonatal period
http://dx.doi.org/10.26452/ijrps.v11i4.3585	Electrolyte imbalances in preterm and term hyperbilirubinemic neonates following phototherapy
http://dx.doi.org/10.1001/jamanetworkopen.2019.0858	Rates of Extreme Neonatal Hyperbilirubinemia and Kernicterus in Children and Adherence to National Guidelines for Screening, Diagnosis, and Treatment in Sweden
http://dx.doi.org/10.1016/j.jpeds.2012.02.018	Glucose-6-phosphate dehydrogenase deficiency and borderline deficiency: Association with neonatal hyperbilirubinemia
http://dx.doi.org/10.1111/j.1442-200X.2007.02507.x	Breast-feeding-associated hypernatremia: Retrospective analysis of 169 term newborns
http://dx.doi.org/10.1056/NEJMoa2020248	Higher or lower hemoglobin transfusion thresholds for preterm infants
http://dx.doi.org/10.1016/j.jpeds.2021.03.057	Effect of Prophylactic Dextrose Gel on Continuous Measures of Neonatal Glycemia: Secondary Analysis of the pre-hPOD Trial
http://dx.doi.org/10.1055/s-0040-1722330	Antenatal Steroids and Acute Kidney Injury in Preterm Infants
http://dx.doi.org/10.1055/s-0037-1604470	Acute Kidney Injury Impairs Postnatal Renal Adaptation and Increases Morbidity and Mortality in Very Low-Birth-Weight Infants
https://www.embase.com/search/results?subaction=viewrecord&id=L2001650047&from=export	Renal functions in term neonates admitted with dehydration in a tertiary care centre in southern Rajasthan
http://dx.doi.org/10.1055/s-2008-1076602	Blood glucose determinations in large for gestational age infants
http://dx.doi.org/10.1001/archpedi.154.11.1140	Prediction and prevention of extreme neonatal hyperbilirubinemia in a mature health maintenance organization
http://dx.doi.org/10.1016/j.amjsurg.2021.03.004	The association between fluid restriction and hyponatremia in newborns with gastroschisis
http://dx.doi.org/10.7860/JCDR/2019/41189.12858	Role of serum cystatin c levels in preterm neonates with respiratory distress syndrome in diagnosing neonatal AKI
http://dx.doi.org/10.3349/ymj.2013.54.4.839	Parenteral nutrition associated cholestasis is earlier, more prolonged and severe in small for gestational age compared with appropriate for gestational age very low birth weight infants

http://dx.doi.org/10.1007/s00467-014-3035-9	Urine neutrophil gelatinase-associated lipocalin in asphyxiated neonates: a prospective cohort study
http://dx.doi.org/10.3233/NPM-200486	Predictability of transcutaneous bilirubinometry in late preterm and term infants at risk for pathological hyperbilirubinemia
http://dx.doi.org/10.1038/pr.2015.25	Serum creatinine concentration in very-low-birth-weight infants from birth to 34-36 wk postmenstrual age
http://dx.doi.org/10.1016/j.jogn.2021.01.007	Transcutaneous Bilirubin Levels and Risk of Significant Hyperbilirubinemia in Early-Term and Term Newborns
http://dx.doi.org/10.1002/jcph.1725	Does “Birth” as an Event Impact Maturation Trajectory of Renal Clearance via Glomerular Filtration? Reexamining Data in Preterm and Full-Term Neonates by Avoiding the Creatinine Bias
http://dx.doi.org/10.1159/000289206	Effect of furosemide on ductal closure and renal function in indomethacin-treated preterm infants during the early neonatal period
http://dx.doi.org/10.1093/tropej/fmw006	Hematological indices at birth of infants of HIV-positive mothers participating in a prevention of mother-to-child transmission program
http://dx.doi.org/10.1038/pr.2016.264	Sodium supply influences plasma sodium concentration and the risks of hyper-and hyponatremia in extremely preterm infants
http://dx.doi.org/10.1038/s41598-019-57334-6	Clinical role of low hemoglobin ratio in poor neurologic outcomes in infants with traumatic intracranial hemorrhage
http://dx.doi.org/10.1016/j.jpeds.2017.09.039	Bilirubin Albumin Binding and Unbound Unconjugated Hyperbilirubinemia in Premature Infants
http://dx.doi.org/10.1136/adc.2009.197699	Perinatal drug exposure and renal function in very preterm infants
http://dx.doi.org/10.1111/tmi.12245	Haematological and biochemical reference values of Gambian infants
http://dx.doi.org/10.4081/nr.2010.e11	BUN:Creatinine ratio - Definition of the normal range in children
http://dx.doi.org/10.1186/s12887-019-1493-8	Predictive value of the aspartate aminotransferase to platelet ratio index for parenteral nutrition associated cholestasis in extremely low birth weight infants
http://dx.doi.org/10.4103/njcp.njcp_368_20	Vitamin D and bone mineral status of newborn-maternal pair delivering at a tertiary hospital in Nigeria
http://dx.doi.org/10.1038/jp.2012.39	Efficacy of phototherapy devices and outcomes among extremely low birth weight infants: Multi-center observational study
http://dx.doi.org/10.1159/000443320	Severe Neonatal Anaemia, MRI Findings and Neurodevelopmental Outcome
http://dx.doi.org/10.1016/S0049-3848(99)00150-4	Effect of iron therapy on the whole blood platelet aggregation in infants with iron deficiency anemia
http://dx.doi.org/10.1007/s00467-018-3899-1	Extra uterine development of preterm kidneys
http://dx.doi.org/10.1038/pr.2013.230	Relationship between acute kidney injury and brain MRI findings in asphyxiated newborns after therapeutic hypothermia
http://dx.doi.org/10.1371/journal.pone.0009033	Does parenteral nutrition influence electrolyte and fluid balance in preterm infants in the first days after birth?
http://dx.doi.org/10.1007/s00467-019-04257-z	Variables of interest to predict glomerular filtration rate in preterm newborns in the first days of life
http://dx.doi.org/10.1056/NEJMoa0803024	Aggressive vs. conservative phototherapy for infants with extremely low birth weight
http://dx.doi.org/10.1542/peds.2013-4299	The natural history of jaundice in predominantly breastfed infants
http://dx.doi.org/10.1055/s-0030-1263301	An observational study of early neonatal biochemical parameters in twins
http://dx.doi.org/10.1371/journal.pone.0196721	Neonatal and maternal serum creatinine levels during the early postnatal period in preterm and term infants
http://dx.doi.org/10.7860/JCDR/2016/18651.7442	Urinary protein creatinine ratio in normal zero to three-day-old Indian neonates
http://dx.doi.org/10.1542/peds.2005-0744	Transcutaneous bilirubin levels in the first 96 hours in a normal newborn population of ≥35 weeks' gestation
http://dx.doi.org/10.3109/14767058.2010.482602	Renal glomerular and tubular function in neonates with perinatal problems
http://dx.doi.org/10.4103/2249-4847.140399	Effect of gestational age, prematurity and birth asphyxia on platelet indices in neonates
https://www.embase.com/search/results?subaction=viewrecord&id=L607893484&from=export	Blood glucose levels within 7 days after birth in preterm infants according to gestational age

http://dx.doi.org/10.1007/s004670000421	Reference intervals for cystatin C in pre- and full-term infants and children
http://dx.doi.org/10.1007/s12098-020-03655-8	Serum Calcium and Melatonin Levels in Neonates Undergoing Phototherapy
http://dx.doi.org/10.1007/s00431-013-2072-x	Blood parameters changes in cord blood of newborns of hypertensive mothers
http://dx.doi.org/10.1136/adc.2005.085449	Procalcitonin in preterm infants during the first few days of life: Introducing an age related nomogram
http://dx.doi.org/10.1159/000074956	Calcium, sodium and potassium urinary excretion during the first five days of life in very preterm infants
https://www.embase.com/search/results?subaction=viewrecord&id=L372656104&from=export	Neonatal haematology in Mauritius - White cells and platelet indices
http://dx.doi.org/10.1515/abm-2020-0010	Prevalence and time course of elevated serum levels of liver enzymes in otherwise healthy Thai infants with breast milk jaundice: A cohort study
http://dx.doi.org/10.1016/j.ekir.2020.09.043	A New Approach to Recognize Neonatal Impaired Kidney Function
http://dx.doi.org/10.1038/s41372-019-0524-5	Renal functional markers in extremely premature infants with and without twin–twin transfusion syndrome
http://dx.doi.org/10.1532/LH96.04076	Hematologic values in healthy and small for gestational age newborns
http://dx.doi.org/10.1111/apa.12041	Reference values of serum cystatin C in very low-birthweight premature infants
http://dx.doi.org/10.1136/archdischild-2015-309246	Lymphocyte subpopulations in premature infants: An observational study
http://dx.doi.org/10.1080/08035250410023179	In vivo spectroscopy of jaundiced newborn skin reveals more than a bilirubin index
http://dx.doi.org/10.1080/14767058.2021.1918667	Reciprocal assessment of urinary beta-2-microglobulin and BUN levels in renal dysfunction of neonates with birth asphyxia
http://dx.doi.org/10.1371/journal.pone.0175936	Human alkaline phosphatase dephosphorylates microbial products and is elevated in preterm neonates with a history of late-onset sepsis
http://dx.doi.org/10.1046/j.1442-200X.2002.01630.x	Nucleated red blood cell counts and erythropoietin levels in high-risk neonates
https://www.embase.com/search/results?subaction=viewrecord&id=L46585449&from=export	Role of procalcitonin, C-reactive protein, interleukin-6, interleukin-8 and tumor necrosis factor-α in the diagnosis of neonatal sepsis
http://dx.doi.org/10.1186/s12887-019-1752-8	First-year growth of 834 preterm infants in a Chinese population: A single-center study
http://dx.doi.org/10.1136/adc.2006.107755	Zinc, copper, selenium and manganese blood levels in preterm infants
http://dx.doi.org/10.1080/14767058.2018.1450858	Parathyroid hormone–reference values and association with other bone metabolism markers in very low birth weight infants–pilot study
https://www.embase.com/search/results?subaction=viewrecord&id=L609360437&from=export	A comparative study of blood glucose measurements using glucometer readings and the standard method in the diagnosis of neonatal hypoglycemia
http://dx.doi.org/10.1016/j.jim.2016.07.001	Flow-based sorting of neonatal lymphocyte populations for transcriptomics analysis
http://dx.doi.org/10.1097/PCC.0000000000001467	Hemoglobin levels across the pediatric critical care spectrum: A point prevalence study
http://dx.doi.org/10.1159/000450674	Reference Ranges of Reticulocyte Haemoglobin Content in Preterm and Term Infants: A Retrospective Analysis
https://www.embase.com/search/results?subaction=viewrecord&id=L364029543&from=export	Prevalence of uridine glucuronosyl transferase 1A1 (UGT1A1) mutations in Malay neonates with severe jaundice
http://dx.doi.org/10.1016/j.ijporl.2020.110126	Audiological assessment of neonatal hyperbilirubinemia
http://dx.doi.org/10.12669/pjms.343.14564	Vitamin B12 deficiency associated with hyperbilirubinemia and cholestasis in infants
http://dx.doi.org/10.1002/14651858.CD013171.pub2	Lipid emulsions for parenterally fed term and late preterm infants
http://dx.doi.org/10.3889/oamjms.2014.043	Incidence of thrombocytopenia in idiopathic hyperbilirubinemic newborns
http://dx.doi.org/10.7196/SAJCH.714	Adherence to phototherapy guidelines in term neonates: Study at a private tertiary-level neonatal unit
http://dx.doi.org/10.2174/1381612824666180918100819	The value of urinary cystatin C level to predict neonatal kidney injury

http://dx.doi.org/10.1111/j.1651-2227.2008.00743.x	Exchange transfusion in infants with extreme hyperbilirubinemia: An experience from a developing country
http://dx.doi.org/10.1038/jp.2015.166	Jaundice, phototherapy and DNA damage in full-term neonates
http://dx.doi.org/10.1159/000358267	A double-blind randomised controlled trial of fish oil-based versus soy-based lipid preparations in the treatment of infants with parenteral nutrition-associated cholestasis
http://dx.doi.org/10.1093/tropej/fmm026	A review of 116 cases of breastfeeding-associated hypernatremia in rural area of central Turkey
http://dx.doi.org/10.1067/mpd.2002.125802	Tauroursodeoxycholic acid (TUDCA) in the prevention of total parenteral nutrition-associated liver disease
http://dx.doi.org/10.1111/apa.12362	The effect of the implementation of therapeutic hypothermia on fluid balance and incidence of hyponatremia in neonates with moderate or severe hypoxic-ischaemic encephalopathy
http://dx.doi.org/10.1002/14651858.CD013171.pub2	Lipid emulsions for parenterally fed term and late preterm infants
http://dx.doi.org/10.5812/ircmj.18288	Evaluation of auditory brain stems evoked response in newborns with pathologic hyperbilirubinemia in Mashhad, Iran
http://dx.doi.org/10.1016/j.bcmd.2012.10.004	Unexplained extreme hyperbilirubinemia among neonates in a multihospital healthcare system
http://dx.doi.org/10.1016/j.ejogrb.2011.02.005	Atazanavir in pregnancy: Impact on neonatal hyperbilirubinemia
http://dx.doi.org/10.1111/j.1440-1754.2006.00975.x	Hypernatraemia in preterm infants born at less than 27 weeks gestation
http://dx.doi.org/10.1097/INF.0b013e31812f56ed	Maternal antiretrovirals and hepatic enzyme, hematologic abnormalities among human immunodeficiency virus type 1-uninfected infants: The NISDI perinatal study
http://dx.doi.org/10.1007/s00467-018-3910-x	Serum glutathione S-transferase Pi as predictor of the outcome and acute kidney injury in premature newborns
http://dx.doi.org/10.3889/MJMS.1857-5773.2014.0356	Incidence of thrombocytopenia in idiopathic hyperbilirubinemic newborns
http://dx.doi.org/10.1038/s41390-018-0249-8	Optimizing the AKI definition during first postnatal week using Assessment of Worldwide Acute Kidney Injury Epidemiology in Neonates (AWAKEN) cohort
http://dx.doi.org/10.1097/MPG.0000000000003103	Applying an Age-Specific Definition to Better Characterize Etiologies and Outcomes in Neonatal Acute Liver Failure
http://dx.doi.org/10.1016/j.cbi.2017.04.014	Association of paraoxonase 1 and oxidative stress with acute kidney injury in premature asphyxiated neonates
http://dx.doi.org/10.3109/14767058.2015.1107900	Acute renal failure in critically ill newborns increases the risk of death: a prospective observational study from India
http://dx.doi.org/10.1111/j.1442-200X.2008.02798.x	Homozygous variant of UGT1A1 gene mutation and severe neonatal hyperbilirubinemia
https://www.embase.com/search/results?subaction=viewrecord&id=L610496650&from=export	Etiologies of prolonged unconjugated hyperbilirubinemia in neonates admitted to neonatal wards
http://dx.doi.org/10.1038/s41390-021-01478-7	Validation of published rebound hyperbilirubinemia risk prediction scores during birth hospitalization after initial phototherapy: a retrospective chart review
https://www.embase.com/search/results?subaction=viewrecord&id=L611581602&from=export	Hepatobiliary scintigraphy for early diagnosis of biliary atresia
http://dx.doi.org/10.1111/j.1442-200X.2008.02577.x	Fluid and electrolyte balance in extremely preterm infants <24 weeks of gestation in the first week of life
http://dx.doi.org/10.3109/14767058.2014.918599	Hypoglycemia incidence and risk factors assessment in hospitalized neonates
http://dx.doi.org/10.1007/s00431-021-03944-0	Conjugated hyperbilirubinemia among infants with hyperinsulinemic hypoglycemia
http://dx.doi.org/10.1111/pan.12512	Sevoflurane anesthesia and brain perfusion
http://dx.doi.org/10.1016/j.cca.2015.08.008	(1)H NMR-based urine metabolic profile of IUGR, LGA, and AGA newborns in the first week of life
http://dx.doi.org/10.1136/adc.2007.135327	Impact of shielding parenteral nutrition from light on routine monitoring of blood glucose and triglyceride levels in preterm neonates
http://dx.doi.org/10.3945/ajcn.111.013938	Effects of iron supplementation on serum hepcidin and serum erythropoietin in low-birth-weight infants
http://dx.doi.org/10.1016/j.earlhumdev.2019.104891	Progressive anemia of prematurity is associated with a critical increase in cerebral oxygen extraction
http://dx.doi.org/10.1111/j.1651-2227.2003.tb00473.x	Role of plasma and urinary calcium and phosphorus measurements in early detection of phosphorus deficiency in very low birthweight infants

http://dx.doi.org/10.1097/JIM.0000000000000222	Serum Sclerostin Levels in Newborns Born to Mothers with Vitamin D Deficiency
http://dx.doi.org/10.1080/080352501317061602	Cord blood levels of cytokines as predictors of early neonatal sepsis
http://dx.doi.org/10.23736/S0026-4946.18.04964-2	Acute kidney injury in preterm neonates with ≤30 weeks of gestational age and its risk factors
http://dx.doi.org/10.1515/JPM.2011.021	Effects of delayed umbilical cord clamping on peripheral blood hematopoietic stem cells in premature neonates
http://dx.doi.org/10.1182/blood-2010-04-278747	Biomarkers of splenic function in infants with sickle cell anemia: Baseline data from the BABYHUG trial
http://dx.doi.org/10.1159/000363729	Duration of gestation and mode of delivery affect the genes of transepithelial sodium transport in pulmonary adaptation
http://dx.doi.org/10.37506/ijphrd.v12i2.14128	Incidence of vitamin d levels in cord blood of newborns and correlation with maternal vitamin D: Our experience
http://dx.doi.org/10.1038/s41390-020-1061-9	Glucocorticoids, sodium transport mediators, and respiratory distress syndrome in preterm infants
http://dx.doi.org/10.1111/jdi.13447	Effects of passage through the digestive tract on incretin secretion: Before and after birth
http://dx.doi.org/10.4049/jimmunol.1601195	Mapping the fetomaternal peripheral immune system at term pregnancy
http://dx.doi.org/10.3109/14767058.2014.927426	Acute funisitis is associated with distinct changes in fetal hematologic profile
http://dx.doi.org/10.1111/sji.12474	Percentiles of Lymphocyte Subsets in Preterm Infants According to Gestational Age Compared to Children and Adolescents
http://dx.doi.org/10.1055/s-2001-16990	Thrombopoietin levels of thrombocytopenic term and preterm newborns with infection
http://dx.doi.org/10.1136/bmjopen-2018-025897	A prospective, cross-sectional study to establish age-specific reference intervals for neonates and children in the setting of clinical biochemistry, immunology and haematology: The HAPPI Kids study protocol
http://dx.doi.org/10.1111/j.1440-1754.2009.01497.x	Impact of delivery mode and gestational age on haematological parameters in Taiwanese preterm infants
http://dx.doi.org/10.1542/peds.2004-2647	Breastfeeding-associated hypernatremia: Are we missing the diagnosis?
http://dx.doi.org/10.7860/JCDR/2017/29137.10303	Changes in hematological parameters in newborns born to preeclamptic mothers - A case control study in a rural hospital
http://dx.doi.org/10.1159/000381330	Reference Intervals of Serum Procalcitonin Are Affected by Postnatal Age in Very Low Birth Weight Infants during the First 60 Days after Birth
http://dx.doi.org/10.1007/s00467-006-0160-0	The urinary activity of angiotensin-converting enzyme in preterm, full-term newborns, and children
http://dx.doi.org/10.1159/000351274	Severe neonatal hyperbilirubinemia in the Netherlands
http://dx.doi.org/10.1007/s12519-009-0057-7	Effect of melatonin on proliferation of neonatal cord blood mononuclear cells
http://dx.doi.org/10.12669/pjms.35.2.342	Neonatal vitamin D status and the risk of neonatal sepsis
http://dx.doi.org/10.1007/s12098-011-0407-z	Efficacy of zinc in reducing hyperbilirubinemia among at-risk neonates: A randomized, double-blind, placebo-controlled trial
http://dx.doi.org/10.1016/S0301-2115(01)00417-1	Short-term outcome of newborn infants: Spinal versus general anesthesia for elective cesarean section: A prospective randomized study
http://dx.doi.org/10.5144/0256-4947.2017.362	Prevalence of glucose-6-phosphate dehydrogenase deficiency in neonates in Egypt
http://dx.doi.org/10.1053/j.semperi.2006.04.001	Kernicterus in Late Preterm Infants Cared for as Term Healthy Infants
http://dx.doi.org/10.1186/s12916-021-01959-w	Novel associations between parental and newborn cord blood metabolic profiles in the Norwegian Mother, Father and Child Cohort Study
http://dx.doi.org/10.1111/apa.14563	Birth weight, Apgar scores and gentamicin were associated with acute kidney injuries in VLBW neonates requiring treatment for patent ductus arteriosus
http://dx.doi.org/10.1097/MPG.0b013e3181c15edf	Copper supplementation in parenteral nutrition of cholestatic infants
http://dx.doi.org/10.1186/s12887-015-0358-z	Management of late-preterm and term infants with hyperbilirubinaemia in resource-constrained settings
http://dx.doi.org/10.1007/s00774-010-0205-0	The influence of folic acid supplementation on maternal and fetal bone turnover

http://dx.doi.org/10.1007/s00467-008-1050-4	Influence of sodium intake on Amphotericin B-induced nephrotoxicity among extremely premature infants
http://dx.doi.org/10.1080/08035250410027634	A high neonatal serum eosinophil cationic protein level is a risk factor for atopic symptoms
http://dx.doi.org/10.1515/jpem-2014-0059	Are preterm newborns who have relative hyperthyrotropinemia at increased risk of brain damage?
